# Understanding Primary Care Dietitians' Experiences and Perspectives on Weight Management Practice Using the COM‐B Model

**DOI:** 10.1111/jhn.70132

**Published:** 2025-09-24

**Authors:** Amira Hassan, Lynne Williams, Megan E. Rollo, Clare E. Collins, Barbara A. Mullan, Hayley Breare, Christina M. Pollard, Deborah A. Kerr, Andrea Begley

**Affiliations:** ^1^ Curtin School of Population Health Curtin University Bentley Western Australia Australia; ^2^ Curtin Medical Research Institute Curtin University Perth Western Australia Australia; ^3^ WA Country Health Service Wheatbelt Western Australia Australia; ^4^ School of Health Sciences, College of Health, Medicine and Wellbeing University of Newcastle Callaghan New South Wales Australia; ^5^ Hunter Medical Research Institute New Lambton Heights New South Wales Australia; ^6^ Enable Institute, Faculty of Health Sciences Curtin University Perth Western Australia Australia

**Keywords:** counselling, dietitians, health behaviour, weight management

## Abstract

**Aim:**

Weight management is a core area of dietetic primary practice. Understanding the behavioural drivers that influence dietitians' weight management practice is essential for gaining insight into how to better support dietitians in this area. This study used the Capability, Opportunity, Motivation‐Behaviour (COM‐B) model, a behavioural framework, to explore how dietitians' practice is influenced by their skills and knowledge (capability), environmental factors (opportunity) and professional desires and habits (motivation).

**Methods:**

This qualitative descriptive study used an interpretivist approach guided by social constructionism. Primary care dietitians practising in Australia were recruited through purposive and snowball sampling for semi‐structured interviews. Interviews were audio‐recorded, transcribed verbatim, and analysed using content analysis. Constructed content categories were then mapped to the COM‐B model to understand the behavioural factors shaping dietitians' weight management practice.

**Results:**

Fourteen primary care dietitians participated in the study; 13 were women, 11 were aged between 20 and 50 years, and 8 had over 9 years dietetic practice experience. Eight content categories were constructed and mapped against the COM‐B components. Aligned with Capability, dietitians demonstrated comprehensive ‘Understanding [of] Weight Management Complexity’ and employed ‘Holistic Practice Approaches’ in their care delivery, though they found ‘Navigating Clients’ Psychosocial Needs’ challenging. Opportunity was shaped by ‘System Constraints’ that influenced care provision, ‘Multidisciplinary Care Environment[s]’ that had varied impacts on collaborative care delivery, and ‘Digital Solutions’ that offered service delivery advantages. Motivation was characterised by dietitians exhibiting strong ‘Professional Drive’ and commitment to ‘Client‐centred Strategies’.

**Conclusion:**

Supporting primary care dietitians requires targeted interventions across all COM‐B components. Potential approaches include enhancing capability through behaviour change counselling training, improving opportunity with integration of digital technologies, and sustaining motivation through established clinical pathways that align with dietitians’ client‐centred values.

## Summary

- First study to systematically map primary care dietitians’ weight management practice experiences using the COM‐B framework, examining how capability, opportunity, and motivation factors shape practice behaviours.
- Identifies key behavioural factors influencing weight management care delivery in primary healthcare settings, including psychosocial counselling capabilities, healthcare system factors, multidisciplinary team dynamics, and digital practice tools.
- Highlights the value of exploring mental health and behaviour change as areas of professional development for dietitians, alongside opportunities to integrate technology into dietetic practice.

## 1 Introduction

Weight management is a multifaceted process reflecting the dynamic relationship between individual‐level factors, like eating patterns and physical activity, and environmental contexts, such as social and economic conditions [1]. Primary healthcare settings often serve as the first point of contact for most individuals seeking weight management support [2, 3, 4] and are positioned as the preferred intervention setting [4, 5]. Primary healthcare intervention approaches for weight management primarily focus on individual‐level change [6]. At the individual level, successful weight management requires sustainable behaviour change, with healthcare professionals supporting clients to modify lifestyle behaviours and eating patterns [7]. Dietitians are well‐positioned for this role, due to their recognised nutrition knowledge and role in facilitating behaviour change [4, 8, 9]. In Australia, weight management represents a significant focus of dietetic practice, accounting for over 50% of consultations, with most of these consultations occurring in primary healthcare settings [10]. Understanding factors influencing dietitians' weight management practice behaviours in Australian primary care settings is therefore important for optimising service delivery and intervention outcomes.

Established contemporary behaviour change frameworks, such as the Capability, Opportunity, Motivation‐Behaviour (COM‐B) model, provide systematic approaches to understanding factors that influence healthcare practice behaviours. Developed by Michie et al., [11] the COM‐B model suggests behaviour is influenced by capability (psychological and physical ability to perform the behaviour), opportunity (physical and social factors that facilitate the behaviour), and motivation (automatic and reflective mechanisms that direct behaviour). The model has demonstrated utility in examining factors influencing healthcare practice behaviours for pharmacists [12, 13, 14], nurses and midwives [15], and primary care professionals, namely medical and nursing practitioners [16, 17]. Among dietitians, COM‐B has been used to examine and facilitate smartphone health application adoption [18, 19] and design workshops targeting dietitians' use of behaviour change techniques [20], yet application to weight management practice remains unexplored. COM‐B applications in weight management contexts primarily focus on patient perspectives around health behaviour change [7, 21, 22]. To our knowledge, only one study has examined COM‐B from a healthcare professional perspective in a weight management context, which explored the experiences of community pharmacists in Malaysia [13].

While some research has explored primary care dietetic practice in Australia more broadly [23, 24, 25, 26, 27], current knowledge of dietetic weight management practice in Australian primary care settings remains limited. Previous studies have examined dietetic weight management practice in acute care [28] and private practice [29] settings, contributing valuable insights, but have not applied behavioural frameworks. There is therefore an opportunity to use such frameworks to systematically explore the factors influencing dietitians' weight management practice behaviours and develop targeted recommendations. The aim of this study is to explore primary care dietitians' weight management practice experiences using the COM‐B model to examine how their capabilities, opportunities, and motivations shape practice behaviours.

## 2 Materials and Methods

The study adopted an interpretivist theoretical perspective to explore dietitians' weight management practice experiences, as this approach was best suited to understanding how dietitians perceive and interpret the various factors affecting their practice [30]. The research was grounded in a social constructionist epistemology, recognising that dietitians' knowledge, meaning, and understanding of their experiences are socially constructed [30]. Focus was placed on how weight management practices are shaped by dietitians' education, professional experiences, client encounters, and broader social contexts, exploring how these interactions produce new knowledge and understanding.

Dietitians' weight management practice was explored through a qualitative descriptive approach, which gathers broad insights into poorly understood experiences, seeking to describe rather than explain them [31]. Semi‐structured online interviews were used to enable flexible, in‐depth exploration of participants' perspectives while increasing accessibility and convenience [32]. The Consolidated Criteria for Reporting Qualitative Research (COREQ) checklist was adhered to and used to present study findings [33]. This study was approved by the Human Research Ethics Committee at Curtin University (HRE2022‐0059).

Study eligibility was determined if interested participants were: (1) dietitians currently practising in Australian primary care settings (e.g. in community, private practice, GP clinics), and (2) providing weight management counselling to clients as part of their current caseload. Participants were recruited through purposive and snowball sampling. Dietitians working in primary care settings across Australia, whose contact information was publicly available on the Dietitians Australia national professional directory [34], were contacted via email and/or phone to assess interest in participating in the study. Digital media advertisements were placed on noticeboards, including Facebook groups for dietetic professionals, Dietitians Australia member communications, and organisational newsletters distributed through primary health networks and private practice groups. Snowballing recruitment occurred through professional networks and participant referrals.

On confirming interest by responding to emails and/or phone calls, individuals were emailed an online screening survey, which contained a participant information sheet. This sheet included information on the interview process, the background of researchers involved, and outlined the interviewer's intention to explore factors influencing dietitians' weight management practices. Participants were required to read the study information and indicate their consent before they could proceed to the demographic and screening questions. Demographic information captured consisted of gender, age, area of residence, and dietetic qualification details. Eligible participants were contacted for interview scheduling and remunerated with a $50AUD gift voucher for participation. A minimum of twelve interviews were planned as it was estimated that this would be appropriate to achieve sufficient data richness and address research questions [35].

The interview guide was iteratively developed through collaboration with dietetic researchers with clinical practice experience and refined after pilot testing with a primary healthcare dietitian recruited through convenience sampling (Table S1). The guide was based on the COM‐B model, examining dietitians' weight management practice through Capabilities (psychological and physical), Opportunities (physical and social), and Motivations (automatic and reflective) [11]. In this application, ‘physical capability’ refers to dietitians' technical consultation skills (e.g. session structuring), rather than physical ability to perform behaviours. Questions were designed to elicit participants' constructed understanding of weight management practice.

Interviews were conducted between December 2022 and March 2023 by Author 2, an experienced senior clinical dietitian and research officer. As professional networks formed part of the recruitment strategy, prior relationships with participants varied. Some participants had no prior contact with the research team, while others were known through limited professional interactions. Interviews were conducted, audio‐recorded, and transcribed using a university‐secure web‐conferencing software (Microsoft Teams). Author 2 kept a reflexive journal during interviews to track assumptions, biases, and evolving perceptions [36]. Regular debriefing between Authors 1, 2, 8, and 9 was undertaken during data collection to discuss emerging patterns and interpretations.

Interviews were conducted until theoretical sufficiency was achieved, where the research team determined that adequate understanding had been gained to address the research question in sufficient depth [37]. After the interviews, member checking was used to allow participants to add or remove details from their transcripts [36], but no participants required any changes. Transcripts were checked for accuracy and managed with NVivo (version 1.7.1, QSR International Pty Ltd).

Author 1 conducted the primary analysis, with ongoing consultation and confirmation from Author 2 throughout. Content analysis was used to examine how dietitians constructed meaning from weight management practice experiences. Analysis involved breaking transcripts into segments that captured participants' experiences of weight management practice [38]. These segments were analysed to generate inductive codes reflecting how dietitians construct meaning around and navigate weight management practice, with repeated reviews enabling progressive refinement of these codes into categories [38]. Categories were synthesised to reflect how behavioural influences shaped dietitians' practice and subsequently mapped to COM‐B components to understand how participants' practice behaviours were reflected in capability, opportunity, and motivation factors. Regular debriefing sessions during analysis involved Authors 1, 2, 8, and 9 reviewing categories, assessing their alignment with the COM‐B model domains, and reaching agreement on COM‐B mapping. All authors reviewed interpretations and COM‐B category mapping to ensure they were well‐supported by the data and appropriately addressed the research question.

## 3 Results

Eighteen individuals expressed interest in participating in an interview. Based on 15 responses provided in the screening survey, 14 were eligible and interviewed. The mean interview duration was 67 min (11.6 SD, range 49–96 min).

Participant characteristics are reported in Table 1. Most participants were women (*n* = 13, (93%), aged between 20 and 50 years (*n* = 11, 79%), with over 9 years since completion of dietetic qualification (*n* = 8, 57%), and based in Western Australia (*n* = 12, 86%). All participants completed their dietetic qualifications in Australia.

**Table 1:** Participant characteristics (*n *= 14).

Gender	
Women	13
Men	1
Age (years)	
20–30	4
31–40	3
41–50	4
51–60	3
Years since completion of dietetic qualification	
9 years or less	6
10–14 years	2
15–19 years	2
20 years or more	4
Primary practice area	
Private practice	4
Local GP practices	3
Regional health services	3
Multi‐service health network	4
Location	
Western Australia	12
South Australia	1
Victoria	1

**Figure 1:**
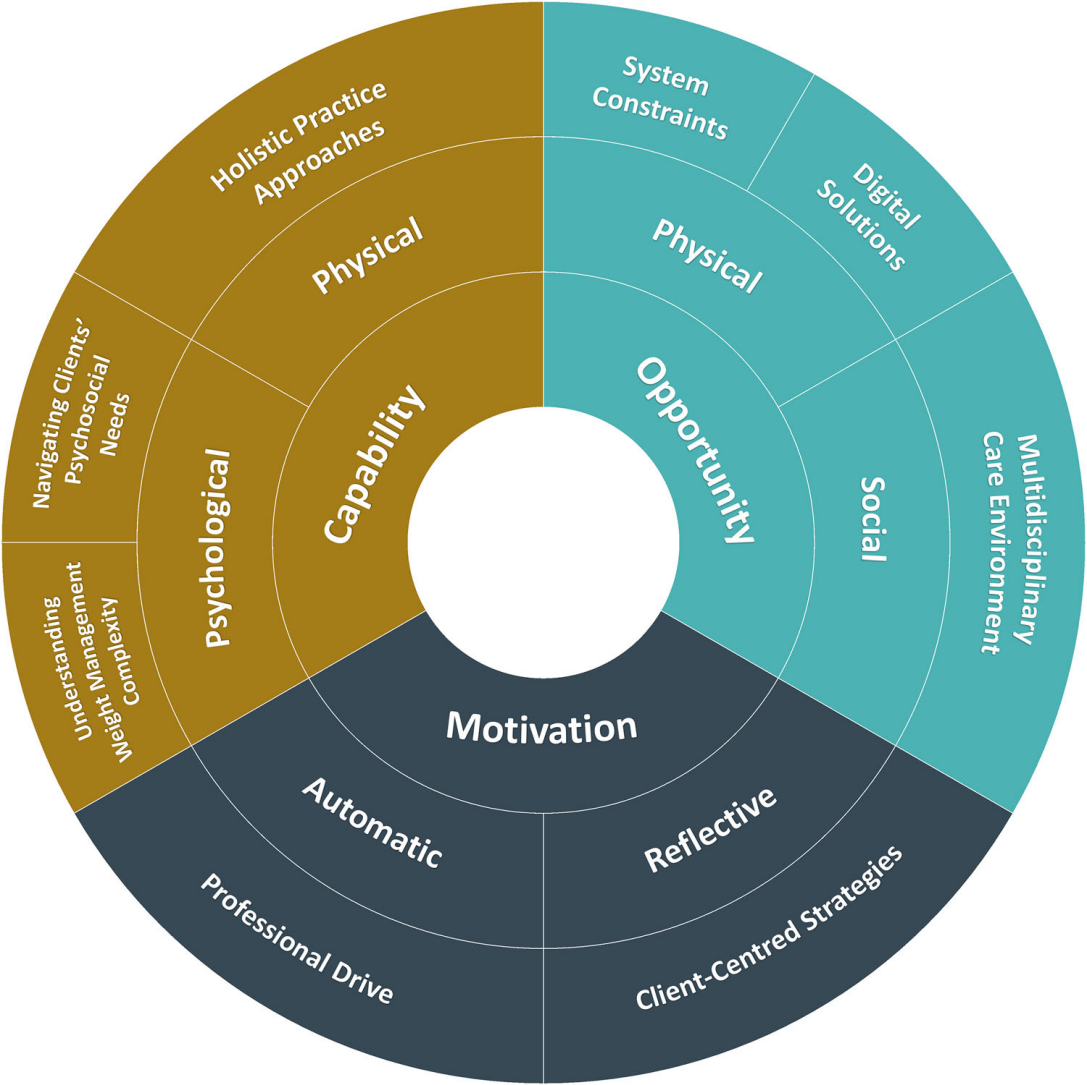
Factors influencing dietitians’ weight management practice mapped to COM‐B components.

Eight categories were constructed and mapped onto the COM‐B model components (Figure 1). Categories are described below, supported by participant quotes from transcripts included to provide insight, with participants identified by a number.

## 4 Capability

### 4.1 Understanding Weight Management Complexity (Psychological Capability)

Psychological Capability involves knowledge and cognitive abilities needed for weight management practice [11]. Two categories were generated within this component. The first category, ‘Understanding Weight Management Complexity,’ reflected dietitians' recognition of the multifaceted nature of weight management and its impact on their practice, with dietitians reporting awareness that clients often faced psychosocial barriers affecting their readiness for behaviour change.

What needs to be fixed first, you know? Her [the client's] social, mental health, psychological [state]? There's all of these other things in the way that would need to be sorted out before we even, you know, look to [weight].

P9

Dietitians stressed that having the knowledge and ability to address these underlying barriers was essential for effective weight management consultations and meaningful behaviour change.

I do say often in jest, if weight loss was easy, I wouldn't have a job. I wouldn't be necessary. But I am necessary because it's so complex. It's not just me telling you what to eat.

P1

### 4.2 Navigating Clients' Psychosocial Needs (Psychological Capability)

The second category, ‘Navigating Clients' Psychosocial Needs,’ highlighted dietitians' challenges in addressing clients' psychosocial aspects in weight management. Several dietitians described their tertiary education as predominantly emphasising biomedical approaches to weight management, revealing knowledge gaps beyond medical nutrition therapy. They noted their education often overlooked the role of psychosocial factors in shaping weight management success, which became evident in their postgraduation practice.

Quite early in my private practice career, I found it frustrating working with clients and doing the old ‘eat less, exercise more’, probably what I'd learned from university.

P12

Despite expressing confidence in their medical nutrition therapy knowledge, some participants described feeling underprepared to support clients and elicit behaviour change, desiring further training to strengthen their capabilities.

It's [weight management] mainly emotional and environmental and social, and all those other things that come with it, and that's where I'm trying to upskill at the moment … because [I've] got the nutrition knowledge. Yep, sound on that. But we need the rest of it too.

P7

### 4.3 Holistic Practice Approach (Physical Capability)

Physical Capability encompasses the practical and technical skills dietitians apply in practice [11]. The category ‘Holistic Practice Approach' was generated within this component, which demonstrated dietitians' preference for weight‐inclusive approaches that encompassed more than dietary advice. Some dietitians made explicit reference to weight‐inclusive frameworks, such as the Health at Every Size® and non‐diet approaches, while most alluded to adopting similar principles in their consultations. In practice, this approach was described as involving examination of eating behaviours and underlying influences to better understand and address factors shaping clients' food choices.

What I'm really interested in is not so much… “what they're eating” at a superficial level. I'm far more interested in looking at what is actually informing the eating choices that they're making.

P13

Dietitians also described avoiding placing sole focus on clients' bodyweight, citing concerns about perpetuating clients' likely historical experiences of weight stigma. Instead, all dietitians emphasised adopting a holistic consultation approach where possible, focusing on *“non‐scale achievements”* (P1).

I will almost always say to them [clients] that weight right now is not our main goal. Our main goal is health and then weight normally comes off with it, so we're not going to obsess over the numbers on the scale.

P4

Dietitians described favouring whole‐food approaches to weight management, promoting mindful and intuitive eating practices instead of restrictive eating plans, which they perceived as less effective for long‐term dietary change.

Any kind of strict meal plan ‐ I don't like. I think it's not giving people the opportunity to really make changes in their own diet… It could very well work for a few weeks and they drop [the weight] all the way. But it's going to come back on because they're not actually making any changes in their lifestyle and in their diet that they're going to stick to.

P4

## 5 Opportunity

### 5.1 *System Constraints* (Physical Opportunity)

Physical Opportunity involves environmental and resource factors affecting practice [11]. Two categories were generated within this component. The first category, ‘System Constraints,’ highlighted the frustration expressed by dietitians with healthcare structures that constrain their weight management consultations and delay weight management support access for clients until after chronic conditions develop.

By the time that they [clients seeking weight‐related care] do get the attention, it's because they've been diagnosed with something, so then they become a higher priority.

P6

Dietitians reported that clients predominantly accessed their services through the Australian Medicare Chronic Disease Management Plan (CDMP), a GP‐referred pathway for individuals with chronic conditions. However, dietitians reported that the limited number of subsidised consultations, which are shared across allied health services, along with restricted consultation length and frequency, restricted their ability to provide comprehensive weight management care. They described being often forced to adjust their care approach to ensure clients left consultations with actionable steps.

Currently, the Medicare system [CDMP] doesn't allow for any more than five allied health visits…which isn't enough… it's not enough for any sort of behaviour change.

P10

We [myself and the client] go physically through this [Eat for Health leaflet], and I put an arrow up if they need to eat more and an arrow down if they need to eat less. Yes, it's prescriptive, but again, time constraints.

P5

### 5.2 *Digital Solutions* (Physical Opportunity)

The second category within Physical Opportunity, ‘Digital Solutions,’ demonstrated technology's role in weight management practice. Dietitians described leveraging digital tools to enhance weight management consultation efficiency. Nearly all dietitians indicated that using dietary tracking applications helped address some time constraints by enabling quicker intake assessment. This was more evident for those who used image‐based dietary assessment, where clients shared captured meal images and dietitians visually evaluated dietary choices and portion sizes.

It makes it a bit easier…for just a quick view to be like, yep…it looks roughly okay [or]…this nutrient might be low or need some extra input.

P3

These dietitians recognised the value of tools that assist with dietary assessment and data collection, noting that comprehensive solutions to streamline assessment processes could allow them to spend more time addressing clients' needs and goals during consultations.

How great would it be if we did have a tool that could kind of collect a lot of that for us?… Because then we can spend more time on the education, and the intervention, and actually digging deeper into more behaviour change.

P11

### 5.3 Multidisciplinary Care Environment (Social Opportunity)

Social Opportunity encompasses interprofessional collaboration and social contexts influencing dietetic practice [11]. The category, ‘Multidisciplinary Care Environment,’ generated within this component, described how dietitians perceived the value of collaborative care despite encountering weight‐stigmatising attitudes from other healthcare providers, particularly GPS. Most reported believing these attitudes contributed to clients' internalised weight stigma, potentially impeding weight management progress.

I'm very aware that…the size of someone's body gets highlighted a lot to people in larger bodies, like…at every doctor's visit…I'm very conscious about how that makes my patient feel, that they've been referred to me because of a health condition, and the doctor's kind of sent them my way to manage their weight first. So, I know that can be very difficult for them.

P7

Most dietitians expressed the belief that weight stigma and biases among healthcare providers, combined with differing discipline‐specific approaches to weight management, obstructed collaborative care delivery.

When you're working with…other allied health professionals…trying to work with them can be really tricky…because they have their own stigmas or biases…as well as the GPs.

P3

Nearly all dietitians indicated that they still valued multidisciplinary weight management care, especially for clients with significant mental health needs. They described addressing these needs as falling outside their scope of practice, which often prompted psychology referrals.

*It's such a complex area. So…yeah, [it] just reinforces the value of having a team approach. I just find, yeah, it's invaluable, particularly psychology.* P8

## 6 Motivation

### 6.1 *Professional Drive* (Automatic Motivation)

Under Automatic Motivation, which includes responses, habits, and intrinsic rewards in practice [11], the category ‘Professional Drive' was generated, capturing dietitians' stated commitment to supporting clients' weight management journeys. This aligned with descriptions of clients' expectations regarding accountability and assistance in progressing toward their weight management goals.

I like people to leave feeling like they've got an ally, and some support, and maybe a new way forward, or at least the knowledge that they have got someone to help them give it their best shot.

P5

Maintaining client motivation and providing reassurance when progress stalled was viewed by dietitians as central to their supportive role, which often involved highlighting that weight management is complex and involves fluctuating progress.

At the end of the day, they just need that one person to guide them, and reassure them, and to let them know that it's a process. It's a process that will have speed bumps…and that's OK…that's human nature. And not to be too disheartened by that, and to just ride the wave.

P2

Many dietitians described using positive reinforcement to acknowledge progress, validate efforts, and enhance clients' sense of accomplishment, which they felt supported maintenance of progress and health behaviour changes.

I find it's a really empowering strategy for them [clients] to have that sense of achievement…like “I've just got a gold star from the dietitian, I'm doing really well, I've got something right all on my own” and they seem to love it.

P1

### 6.2 Client‐Centred Strategies (Reflective Motivation)

Under reflective Motivation, which explores conscious decisions about approaches to support clients seeking weight‐related care [11], the category ‘Client‐Centred Strategies' was generated. This category demonstrated dietitians’ expressed commitment to client‐centred care through tailored approaches to support clients' behaviour change. During initial consultations, dietitians perceived clients' anticipation of weight stigma when clients hesitated discussing weight concerns or pre‐emptively justified dietary behaviours. This perception was suggested to stem from clients' prior experiences of weight stigma with healthcare professionals. Dietitians agreed that establishing therapeutic rapport should be prioritised over dietary advice delivery, as trusting relationships were described as essential for facilitating behaviour change.

What is important for me in the first session is building that rapport and laying the groundwork for a strong therapeutic alliance. Establishing trust is really important…so it may not look [like] medical nutrition therapy at all.

P13

All dietitians described tailoring practice to each client's circumstance and change readiness, setting personalised, achievable goals collaboratively to encourage meaningful engagement in behaviour change. Motivational interviewing was also described as being used to explore clients' underlying reasons for pursuing weight management and reinforce long‐term behaviour change.

Apply[ing] a “one shoe fits all” sort of application can potentially set things up to fail.

P9

I generally have my patients complete like a values clarification because, without the motivation and the drive to change, I don't see the point in giving surface‐level recommendations that people probably won't implement.

P14

## 7 Discussion

Through the lens of the COM‐B model, this study revealed how primary care dietitians practising in Australia construct and navigate weight management practice. These findings highlight a disparity between dietitians' understanding of weight management complexity and perceived capability to address underlying psychosocial factors of behaviour change. Healthcare system constraints created practice barriers, prompting dietitians to leverage multidisciplinary collaboration and digital solutions. Despite barriers, dietitians demonstrated motivational drivers through commitment to client‐centred approaches. These interconnected factors shape dietitians' construction of weight management practice, revealing opportunities to further strengthen practice.

Dietetic interventions for health behaviour change are essential for sustainable weight management in primary care settings [39]. The Nutrition Care Process, a systematic framework used globally by dietitians to guide the delivery of nutrition care [40], inherently supports applying behaviour change techniques in dietetic practice [41]. Despite this embedded approach, dietitians in this study identified significant behavioural change training gaps. This is consistent with findings from Australian dietetic surveys examining weight management practice across diverse dietetic practice settings over the past two decades [10, 42]. This perceived competency gap may be attributable to several factors. Most participants completed their dietetic qualifications in Australia over 9 years ago under previous competency standards that exhibited variations in how behaviour change training was integrated across Australian tertiary institutions [43]. The National Competency Standards for Dietitians in Australia were revised in 2020 to include behaviour change competencies, requiring dietitians to apply behavioural science knowledge into practice [44]. Additionally, private dietetic practice employment has experienced rapid growth [45], resulting in longer‐term dietitian‐client relationships compared to public health settings, potentially creating increased demands for behaviour change competencies. Further research is needed to evaluate whether the updated competency standards have been effectively implemented in dietetic curricula and examine professional development opportunities to support dietitians' behaviour change capabilities.

The training gap in health behaviour change is further complicated by prevalent mental health challenges among clients seeking weight‐related care. Dietitians frequently work with clients experiencing mental health challenges [46], and although dietary interventions can improve mental health outcomes [47], mental health status represents a substantial barrier to undertaking health behaviour change [48]. Despite recognising this impact on clients' capacity for dietary behaviour change, many participants in our sample felt ill‐equipped to navigate mental health complexities effectively, often referring clients to mental health services. The consistency of this practice pattern across participants may reflect gaps in professional competency requirements. Mental health competency is not explicitly emphasised in the current National Competency Standards for Dietitians in Australia [49], except for those specifically working in mental health settings [50]. Our findings suggest there may be value in exploring whether competency standards could expand to emphasise mental health response skills for all dietitians, particularly where mental health influences eating behaviours [46]. Larger‐scale, mixed‐methods studies involving dietitians from diverse practice settings would be valuable to validate these preliminary findings and determine the broader need for enhanced mental health competencies among dietitians in Australia.

Weight stigma presents another practice challenge for dietitians. Defined as “discriminatory acts and ideologies targeted towards individuals because of their weight and size” [51], weight stigma is pervasively experienced by individuals with higher weight, particularly in healthcare settings [52, 53, 54], and is associated with adverse mental health outcomes and health behaviours [55]. Growing recognition of these harms, alongside evidence of the limitations of weight‐focused interventions, has prompted development of alternative weight‐inclusive approaches that prioritise health behaviours regardless of body size, such as the Health at Every Size® and non‐diet approaches [1, 56]. Reflecting this shift, dietitians in our sample favoured consultation approaches that emphasised behavioural changes and overall health improvements, consistent with findings in studies of other groups of dietitians working with individuals with higher weight [57, 58, 59]. Our participants, however, also described how weight‐stigmatising attitudes from other healthcare providers could undermine these weight‐inclusive efforts and reinforce clients' internalised weight stigma. This creates a tension between the recognised value of multidisciplinary approaches to weight management [60, 61], and the challenges that arise when team members operate from different paradigms regarding weight and health. Evidence demonstrates that weight‐inclusive approaches in multidisciplinary settings can improve health behaviours and psychological outcomes [1, 62], with research showing these approaches achieve comparable and better health outcomes than weight‐centric interventions [63]. This presents an opportunity to examine how aligned approaches within primary care weight management teams might improve the effectiveness of their support.

Dietitians face significant systemic constraints in providing comprehensive care, particularly within the CDMP. Dietetic services have been the third most utilised allied health service under the CDMP pathway for over 20 years [64, 65, 66]. However, the CDMP provides only five annual consultations, which must be shared among various allied health professionals [67]. The currently rescinded Australian weight management guidelines, which continue to influence current practice pending updated versions, recommended regular consultations during initial weight loss with continued monitoring for at least 12 months [4]. This substantial support gap between available and recommended care undermines sustainable behaviour change, which demands consistent guidance and reinforcement over extended periods [68]. The CDMP's prerequisite of established chronic conditions further constrains practice by compelling dietitians to adopt a reactive approach focused on managing adiposity‐related complications rather than preventive interventions. These time and resource constraints resonate as a widespread concern among dietitians [26, 69, 70, 71], highlighting the need for a comprehensive clinical care pathway for individuals with higher weight. Such a pathway should align with evidence‐based recommendations for frequent, long‐term support [5] to facilitate behaviour change in clients seeking weight‐related care, medical management, or both.

Dietitians are using technological solutions to enhance and bridge gaps in dietetic practice [72]. Approximately half of the interviewed dietitians have incorporated dietary assessment technologies into their practices, including web‐based tools and mobile apps. These digital tools enable systematic collection and analysis of dietary intake data through electronic records or image‐based logging [73], enhancing nutritional assessment precision while freeing consultation time for weight management counselling [74]. These assessments can improve eating behaviours and bodyweight outcomes when paired with personalised dietary feedback [75]. Despite these benefits, use of digital tools in dietetic practice predominantly facilitates client self‐monitoring and information delivery, representing underutilised potential to deliver comprehensive behaviour change interventions [18]. Better leveraging of digital tools in dietetic practice for intervention delivery, such as through automated feedback systems [18], could help dietitians optimise resource management while providing the timely support clients need for successful behaviour change.

Our findings offer insights into dietitians' experiences with weight management practice, acknowledging that these understandings are co‐constructed through researcher‐participant interactions and participants' particular contexts and perspectives. The research team's reflexive practice enhanced the credibility and confirmability of interpretations. While participants were predominantly women, this aligns with the demographics of the wider dietetic profession [76]. The overrepresentation of Western Australian dietitians limits generalisability to other Australian states and territories, though significant variation in dietetic weight management practice across Australia is unlikely. Additionally, our focus on primary care weight management may not represent the diverse weight management practice experiences of dietitians working in other practice settings.

## 8 Conclusion

This study reveals a complex interplay of factors influencing primary care dietitians' weight management practice through the COM‐B lens. While dietitians demonstrate strong motivation for client‐centred care, they face significant practice barriers due to perceived behaviour change skill gaps and systemic constraints. These findings highlight several areas for future investigation: evaluating the implementation of updated competency standards in dietetic education and professional development; conducting larger‐scale validation studies to determine mental health competency needs among dietitians practising in Australia; exploring strategies for multidisciplinary team alignment in primary healthcare weight management; and assessing the effectiveness of technology‐enhanced interventions in addressing service delivery barriers. Alongside these future research directions, the specific insights from this study will inform the development of training approaches and intervention implementation protocols for a dietitian‐led digital weight management study involving primary care dietitians.

## Author Contributions

All authors participated in the conceptualisation and methodology of the study. Lynne Williams conducted the data collection. Amira Hassan conducted the primary analysis with ongoing consultation from Lynne Williams, with all authors contributing to the interpretation of the data. Amira Hassan was responsible for data curation and writing the original draft. All authors critically revised the manuscript and read and approved the final version.

## Conflicts of Interest

The authors declare no conflicts of interest.

## Peer Review

The peer review history for this article is available at https://www.webofscience.com/api/gateway/wos/peer-review/10.1111/jhn.70132.

## Supporting information


**Supplementary Table 1:** Interview Guide for Dietitians with Questions Mapped to COM‐B Components.

## Data Availability

The data that support the findings of this study are available from the corresponding author upon reasonable request.
