# Supplementary material for: Understanding Primary Care Dietitians' Experiences and Perspectives on Weight Management Practice Using the COM‐B Model
Source: J Hum Nutr Diet. 2025 Sep 24;38(5):e70132. doi: 10.1111/jhn.70132 (PMC12459232; doi:10.1111/jhn.70132)
Supplement: Supplementary file 1 — Supplementary Table 1: Interview Guide for Dietitians with Questions Mapped to COM‐B Components. [file JHN-38-0-s001.docx]

| **Supplementary Table 1. Interview Guide for Dietitians with Questions Mapped to COM-B Components** | |
| --- | --- |
| **COM-B Model Categories** | **Related Interview Questions** |
| **Capability** | |
| **Psychological Capability**  Explores participants' knowledge, skills, and cognitive abilities for dietetic weight management practice | What factors do you find most commonly influence your client's ability to make diet and behaviour changes?  Could you describe any formal weight management training you have completed?  How do you perceive or measure success for weight management? |
| **Physical Capability***  Explores the practical and technical skills used by dietitians during weight management practice | How do you approach weight management consultations?  Can you describe the dietary advice you typically provide? |
| **Opportunity** | |
| **Physical Opportunity**  Explores environmental and resource factors affecting dietetic weight management practice | What challenges do you face in helping clients with weight management?  What have been your experiences using different consultation delivery methods with clients (e.g. telehealth, face-to-face, over the phone)?  What dietary assessment tools do you rely on in practice? |
| **Social Opportunity**  Explores the interprofessional and social factors influencing dietetic weight management practice | Could you share any experiences where you have encountered weight stigma for your clients or in the workplace? |
| **Motivation** | |
| **Automatic Motivation**  Explores dietitians’ responses, habits, and intrinsic rewards associated with delivering weight management practice | How do clients rely on you to change dietary behaviour?  When clients seem unmotivated to make changes, how do you typically approach these situations? |
| **Reflective Motivation**  Explores conscious and deliberate decisions dietitians made about approaches used to support clients seeking weight-related care | Can you describe your approach during an initial consultation with a client seeking weight management support?  What behaviour change techniques do you find most effective to help clients create sustainable behaviour changes? |
| *In this application of the COM-B model, 'physical capability' has been interpreted as the practical and technical skills employed by dietitians during weight management practice (e.g., session structuring), rather than the physical ability to perform behaviours. | |
